# Supplementary figures and images for: Translation of zinc finger domains induces ribosome collision and Znf598-dependent mRNA decay in zebrafish
Source: PLoS Biol. 2024 Dec 5;22(12):e3002887. doi: 10.1371/journal.pbio.3002887 (PMC11620358; doi:10.1371/journal.pbio.3002887)

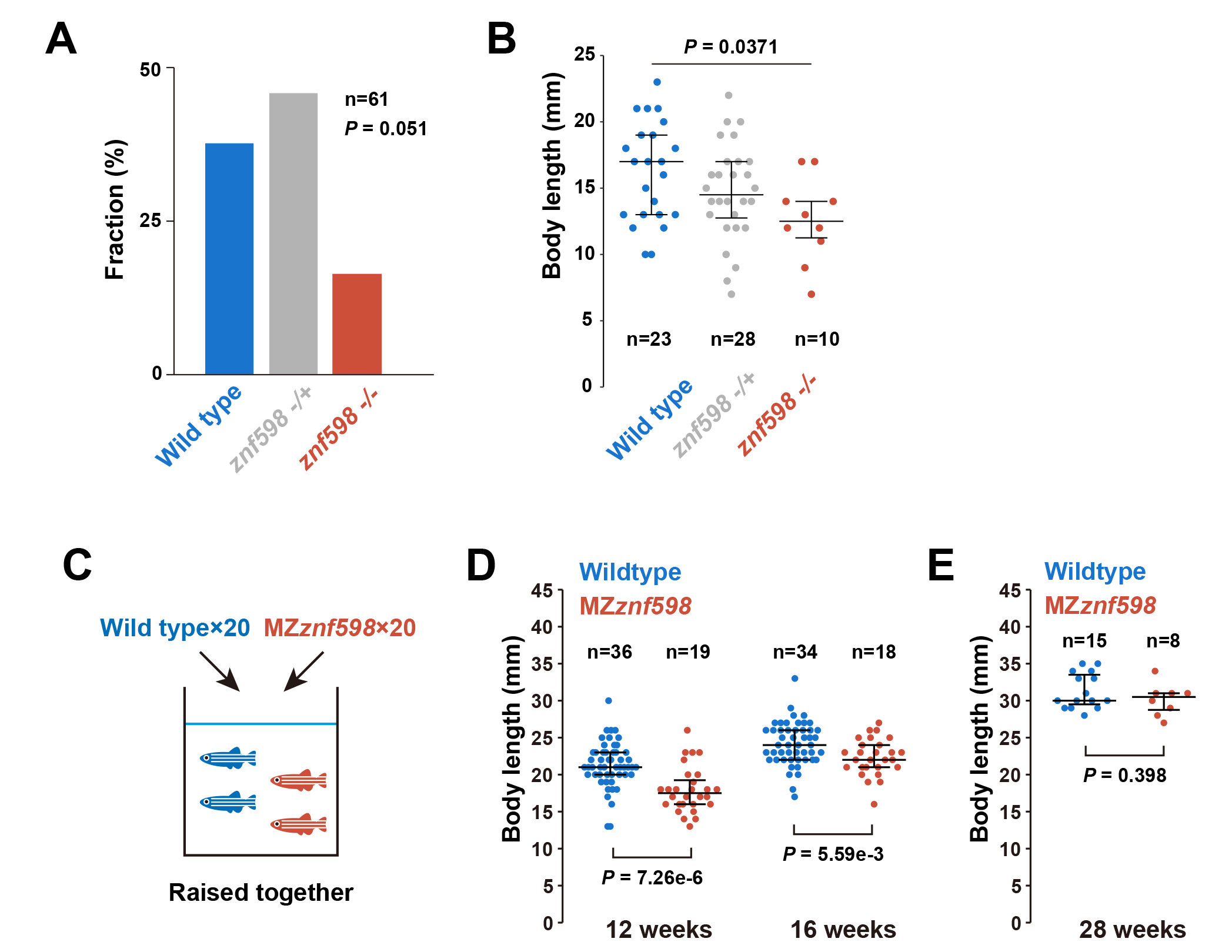

Supplement: S1 Fig — (A) The genotyping results of 10-week-old siblings obtained by crossing heterozygous znf598 mutant fish. The p value was calculated by the chi-square test. (B) Distributions of body length in 10-week-old wild-type, znf598 heterozygous, and znf598 homozygous sibling fish obtained by crossing heterozygous znf598 mutant fish. The p values were calculated by one-way ANOVA test. (C) A scheme of the growth and survival test comparing wild-type and MZznf598 fish. (D, E) Distributions of the body length in wild-type and MZznf598 fish at 12, 16, and 28 weeks after birth. In (D), results of the 2 experiments were combined and plotted. In (E), results of a single experiment were plotted. The p values were calculated by the Mann–Whitney U test (two-tailed). The data underlying this figure can be found in S1 Data. (TIF) [file pbio.3002887.s001.tif]

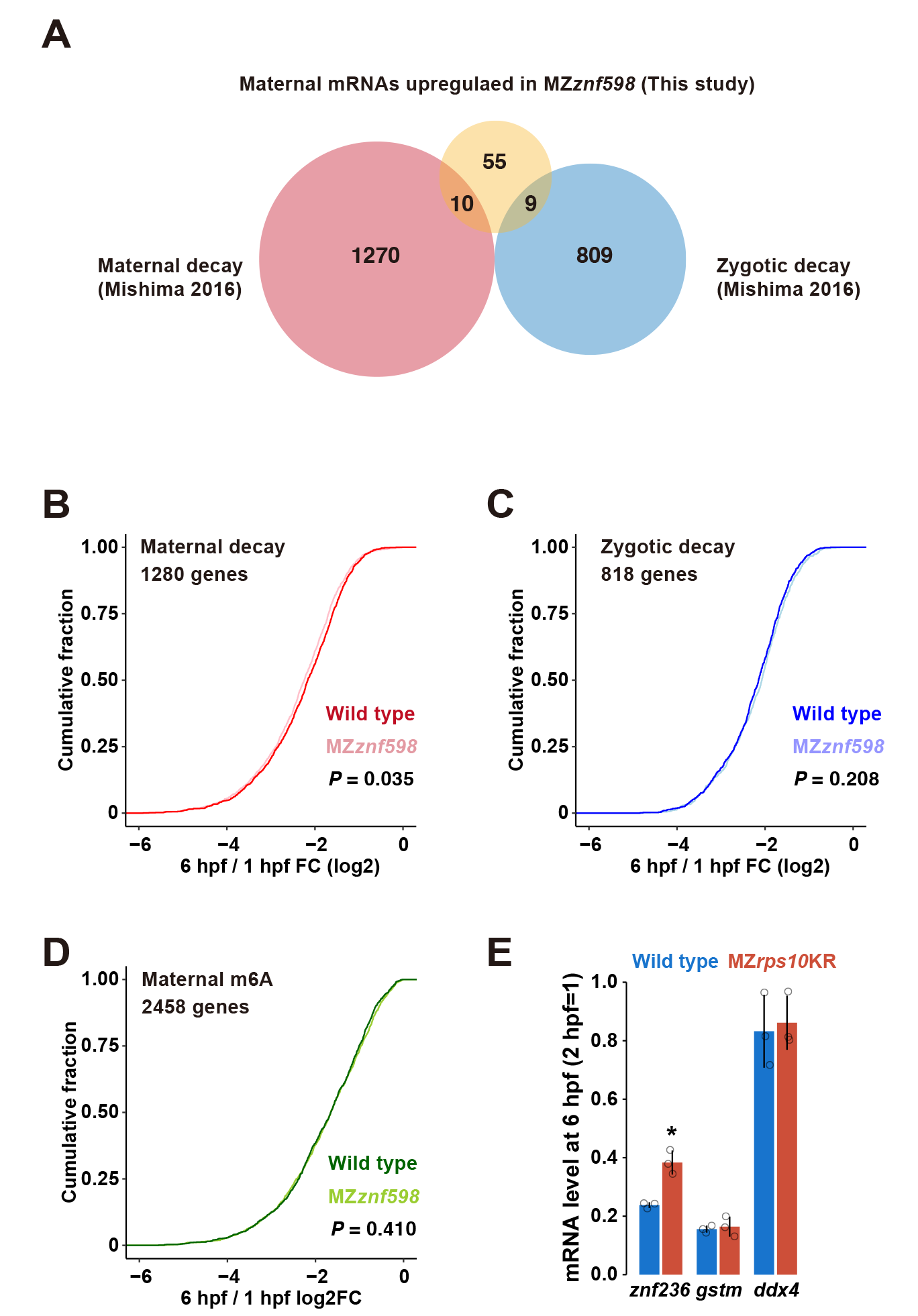

Supplement: S2 Fig — (A) A Venn diagram of NGD target candidates (yellow), maternal decay mRNAs (pink), and zygotic decay mRNAs (light blue). The numbers of genes in each category are shown. (B–D) Cumulative distributions of maternal decay mRNAs, zygotic decay mRNAs, and maternal mRNAs with a high level of m6A modification. The x-axis shows the fold change in mRNA expression at 6 hpf compared to 1 hpf in wild-type and MZznf598 embryos, and the y-axis shows the cumulative fraction. The p values calculated by the Kolmogorov–Smirnov test are indicated. (E) qRT-PCR analysis of znf236 mRNA in wild-type (blue) and MZrps10KR embryos (red) at 6 hpf relative to 2 hpf. gstm mRNA (miR-430 target) and ddx4 mRNA (stable maternal mRNA) are shown as controls. The asterisk indicates p < 0.05 (Student’s t test). The data underlying this figure can be found in S1 Data. (TIF) [file pbio.3002887.s002.tif]

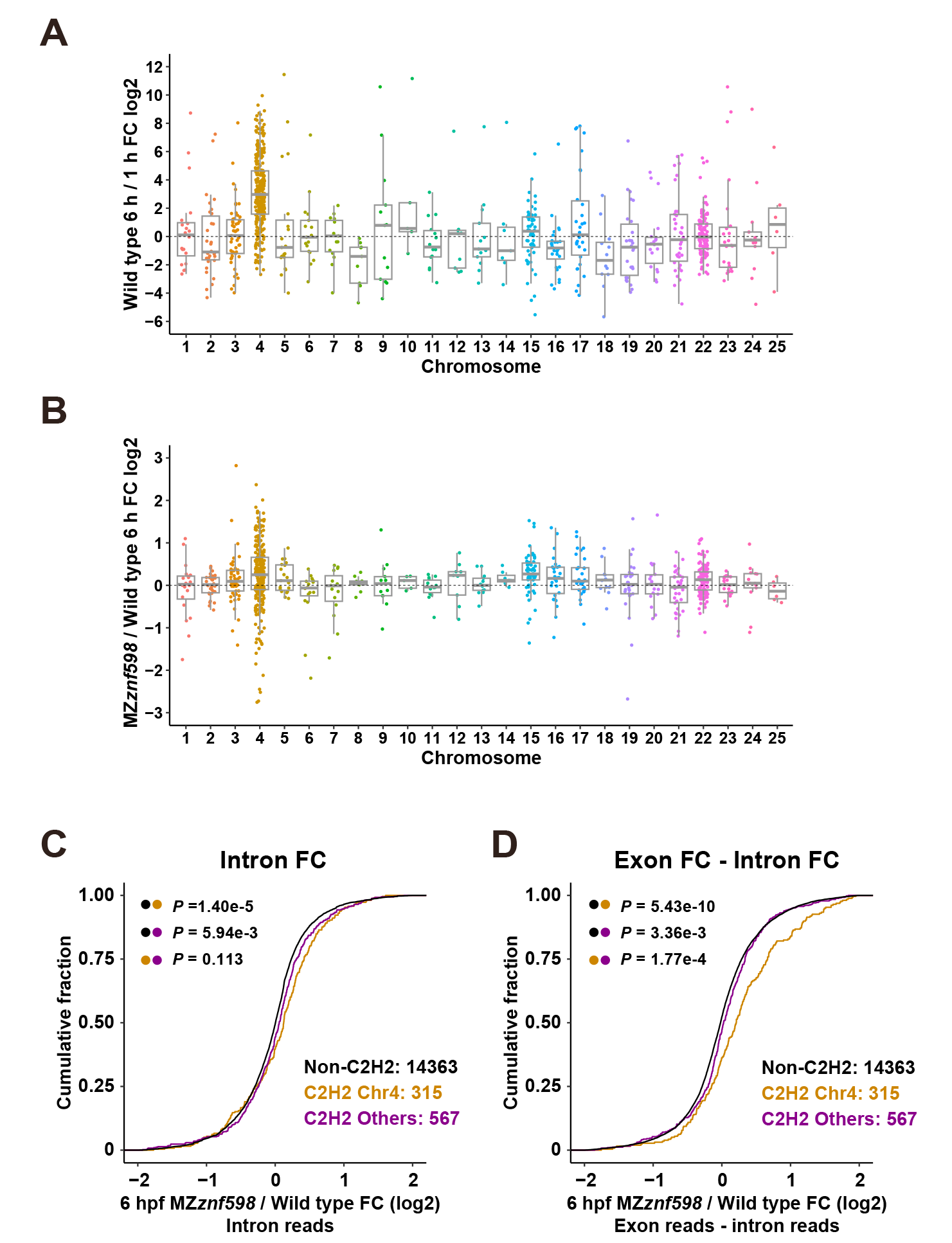

Supplement: S3 Fig — (A) Chromosomal distributions of C2H2-ZF genes and their relative mRNA expression at 6 hpf compared to 1 hpf in wild-type embryos. The x-axis shows chromosome numbers, and the y-axis shows the fold change in mRNA levels at 6 hpf compared to 1 hpf. (B) Chromosomal distributions of C2H2-ZF genes and their relative mRNA expression in MZznf598 embryos compared to wild-type embryos at 6 hpf. The x-axis shows chromosome numbers, and the y-axis shows fold changes in mRNA levels. In A and B, the value of each C2H2-ZF gene is plotted as a dot. The box represents the interquartile range (IQR), with the median indicated by the thick horizontal line in the box. The whiskers represent the variation within 1.5 IQR outside the upper and lower quartiles. (C) Cumulative distributions of fold changes in intron RNA levels in MZznf598 embryos compared to wild-type embryos at 6 hpf. (D) Cumulative distributions of the mature mRNA fold change subtracted by the intron RNA fold change in MZznf598 embryos compared to wild-type embryos at 6 hpf. In C and D, C2H2-ZF genes on chromosome 4 (orange), C2H2-ZF genes on other chromosomes (purple), and genes without C2H2-ZF (black) are shown. The x-axis shows the fold change, and the y-axis shows the cumulative fraction. The p values are shown on the left (Kolmogorov–Smirnov test). The data underlying this figure can be found in S1 Data. (TIF) [file pbio.3002887.s003.tif]

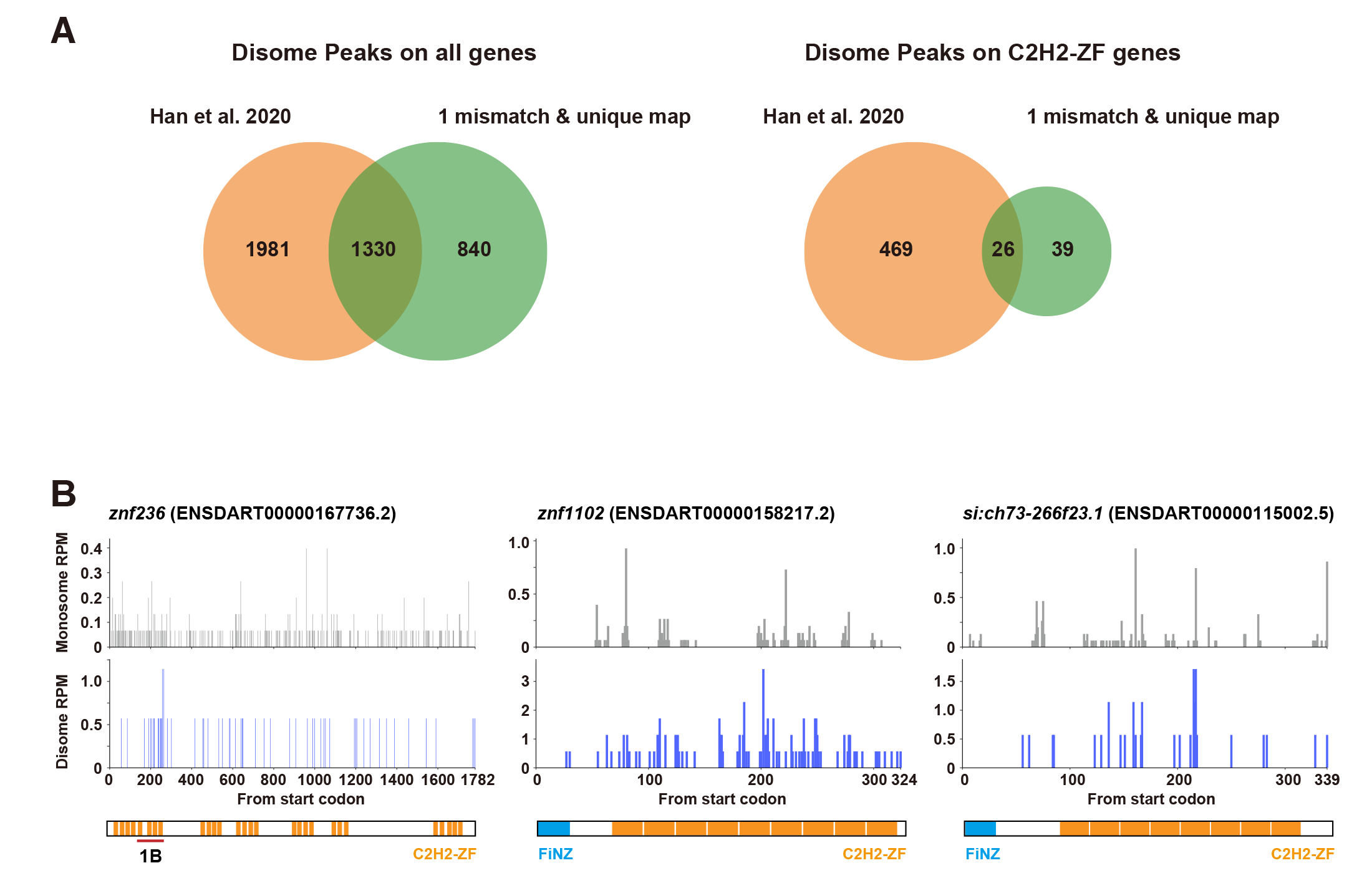

Supplement: S4 Fig — (A) Venn diagrams of disome peaks detected in a previous study [41] and in this study (1 mismatch and unique map). The number of peaks detected in each study is shown. Left: disome peaks in all genes. Right: disome peaks in C2H2-ZF genes. (B) Distributions of monosome (upper, gray) and disome (lower, blue) footprints on the znf236, znf1102, and si:ch73-266f23.1 ORFs. The data underlying this figure can be found in S1 Data. (TIF) [file pbio.3002887.s004.tif]

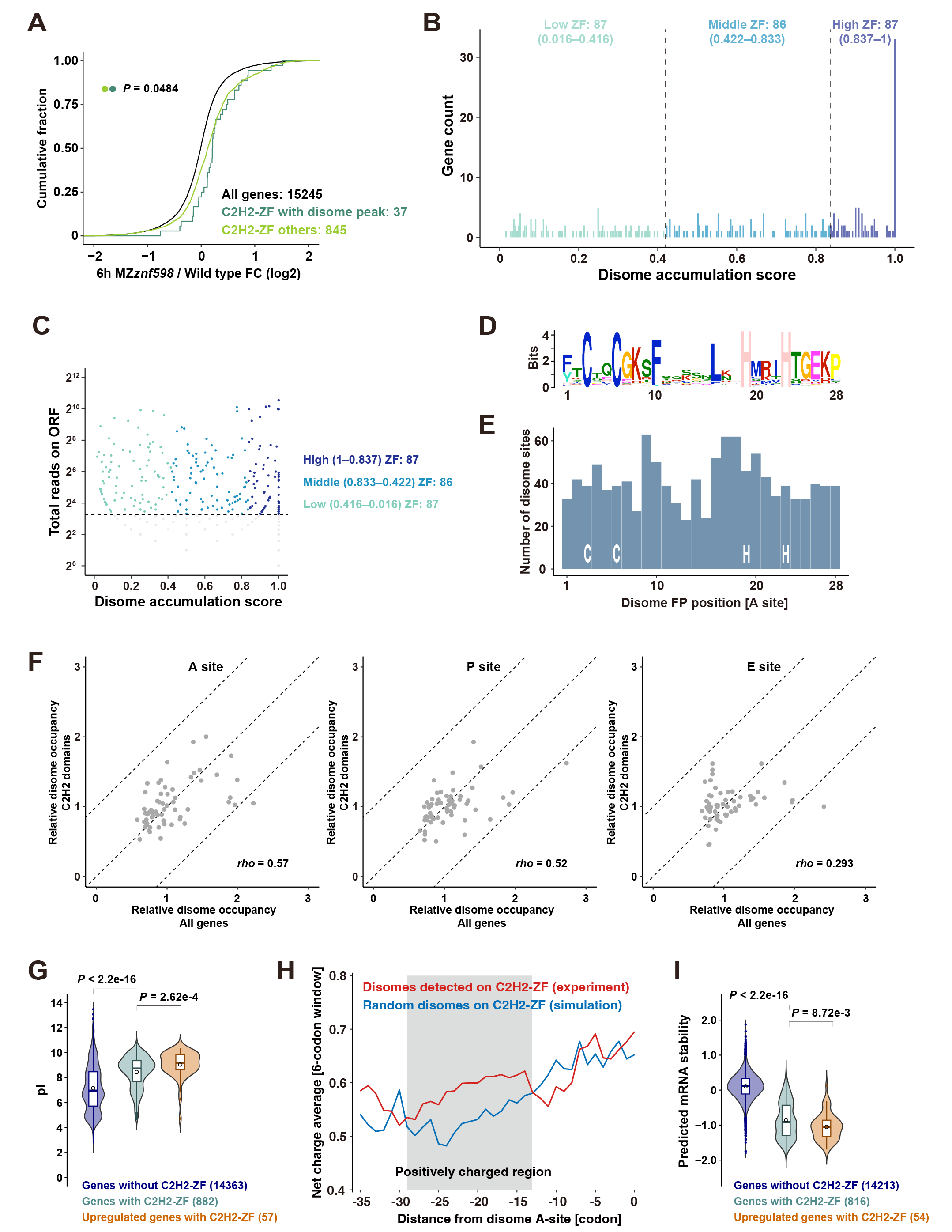

Supplement: S5 Fig — (A) Cumulative distributions of fold changes in mRNA levels in MZznf598 embryos compared to wild-type embryos at 6 hpf. All genes (black) and C2H2-ZF genes with disome peaks (green) or without disome peaks (light green) are shown. The x-axis shows the fold change, and the y-axis shows the cumulative fraction. The p values are shown on the left (Kolmogorov–Smirnov test). (B) A histogram showing the distributions of disome localization scores on C2H2-ZF genes. The x-axis shows the disome localization score, and the y-axis shows the gene count. (C) A scatter plot showing the disome localization score (x-axis) and total read number for each ORF (y-axis). (D) A logo representation of amino acid diversity among 8,168 C2H2-ZF proteins collected from zebrafish genes. (E) A histogram showing the A-site position of the leading ribosome in disome footprints relative to the C2H2-ZF sequence in zebrafish. (F) Disome occupancy at the A-, P-, or E-site codon of the leading stalled ribosome in all genes (x-axis) and C2H2 sequences (y-axis). rho, Spearman’s rank correlation. (G) Violin plots showing the distribution of the predicted pI values in zebrafish genes. pI values of genes without C2H2-ZF (blue), genes with C2H2-ZF (turquoise), and C2H2-ZF genes up-regulated in MZznf598 (orange) are shown. (H) Mean net charge in the nascent chain around ribosome collision sites on C2H2-ZF sequences in zebrafish. Nascent chain sequences determined by the A-site position of experimentally detected disome footprints (red) and those determined by the randomly chosen A-site position (blue) are shown. (I) Violin plots showing the distribution of the predicted mRNA stability by iCodon in zebrafish genes. The predicted mRNA stabilities of genes without C2H2-ZF (blue), genes with C2H2-ZF (turquoise), and C2H2-ZF genes up-regulated in MZznf598 (orange) are shown. The p values in G and I were calculated by the Wilcoxon rank sum test. The data underlying this figure can be found in S1 Data. (TIF) [file pbio.3002887.s005.tif]

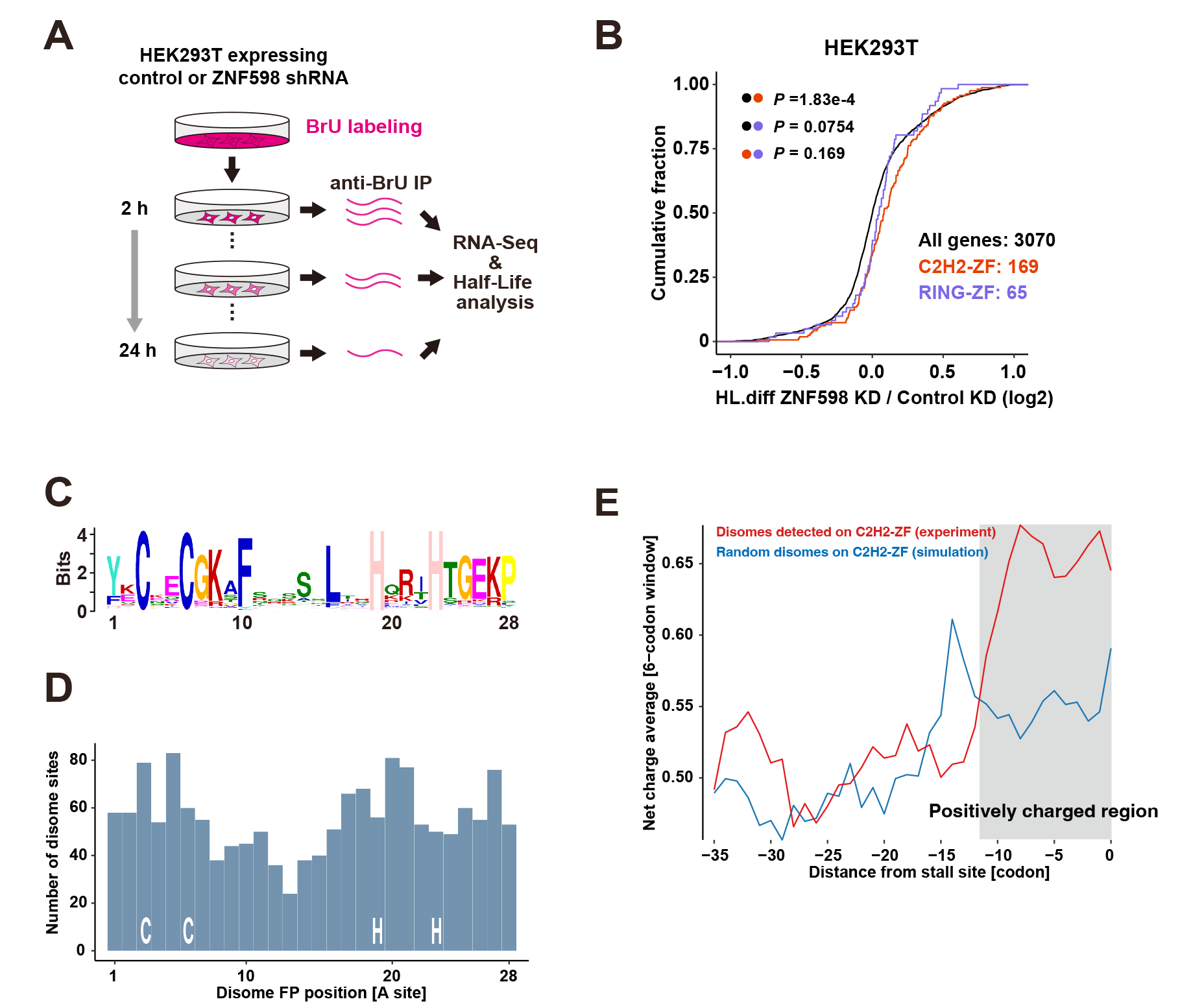

Supplement: S6 Fig — (A) A schematic of BRIC-Seq in HEK293T cells. (B) Cumulative distributions of fold changes in the mRNA half-life in HEK293T cells, comparing ZNF598 knockdown cells and control cells. All genes (black), C2H2-ZF genes (red), and RING-ZF genes (purple) are shown. The x-axis shows the fold changes in the mRNA half-life. The p values are shown on the left (Kolmogorov–Smirnov test). (C) A logo representation of amino acid diversity among C2H2-ZF collected from human genes. (D) A histogram showing the A-site position of the leading stalled ribosome in disome footprints relative to the C2H2-ZF sequence in HEK293 cells. (E) Mean net charge in the nascent chains around ribosome collision sites on C2H2-ZF sequences in HEK293 cells. Nascent chain sequences determined by the A-site position of experimentally detected disome footprints (red) and those determined by the randomly chosen A-site position (blue) are shown. The data underlying this figure can be found in S1 Data. (TIF) [file pbio.3002887.s006.tif]

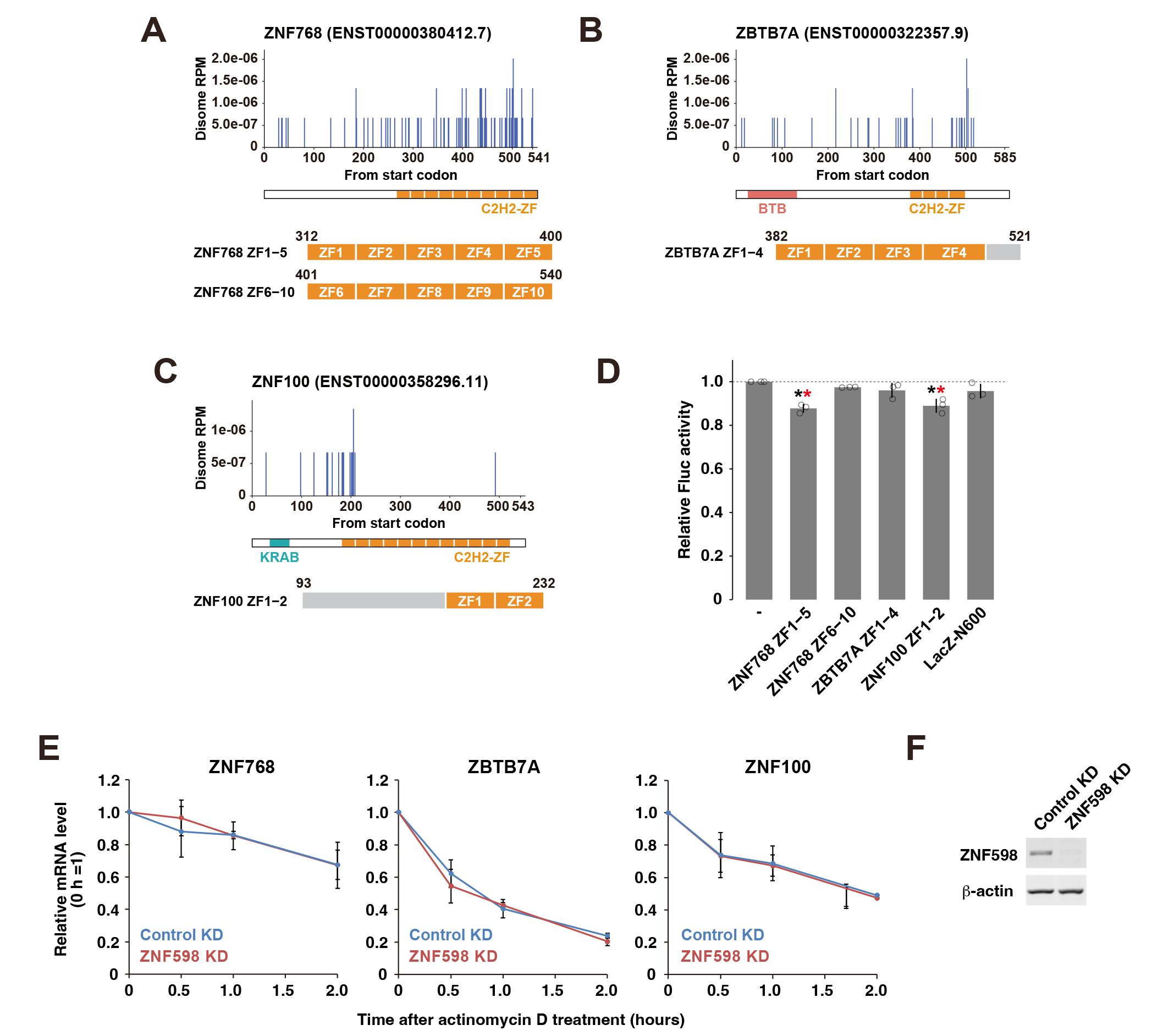

Supplement: S7 Fig — (A–C) Distributions of disome footprints (blue) in the ZNF768, ZBTB7A, and ZNF100 ORFs. C2H2-ZF is indicated in orange. The fragments encoding C2H2-ZF repeats with high disome footprint amounts used for validation experiments are shown below with amino acid positions. (D) Results of the tandem ORF assay with human C2H2-ZF sequences in HEK293T cells. The graphs represent the average of 3 independent experiments. Relative Fluc signals normalized to Rluc signals are shown. Values with no insert (-) were set to one. The error bars show the standard deviation. The open circles show each data point. Black asterisks indicate p < 0.05 compared to no insert. Red asterisks indicate p < 0.05 compared to LacZ-N600 (Dunnett’s test). (E) Time course qRT-PCR analysis of ZNF768, ZBTB7A, and ZNF100 mRNAs after Actinomycin D treatment in control (blue) and ZNF598 knockdown (red) HEK293T cells. The graphs represent the average of 3 independent experiments. The error bars show the standard deviation. (F) Western blotting to detect ZNF598 proteins in control and ZNF598 knockdown cells. β-actin was detected as a loading control. The data underlying this figure can be found in S1 Data. (TIF) [file pbio.3002887.s007.tif]
