# Supplementary material for: Translation of zinc finger domains induces ribosome collision and Znf598-dependent mRNA decay in zebrafish
Source: PLoS Biol. 2024 Dec 5;22(12):e3002887. doi: 10.1371/journal.pbio.3002887 (PMC11620358; doi:10.1371/journal.pbio.3002887)

Figure 1B raw data

Lane1: wild type, Lane2: MZznf598

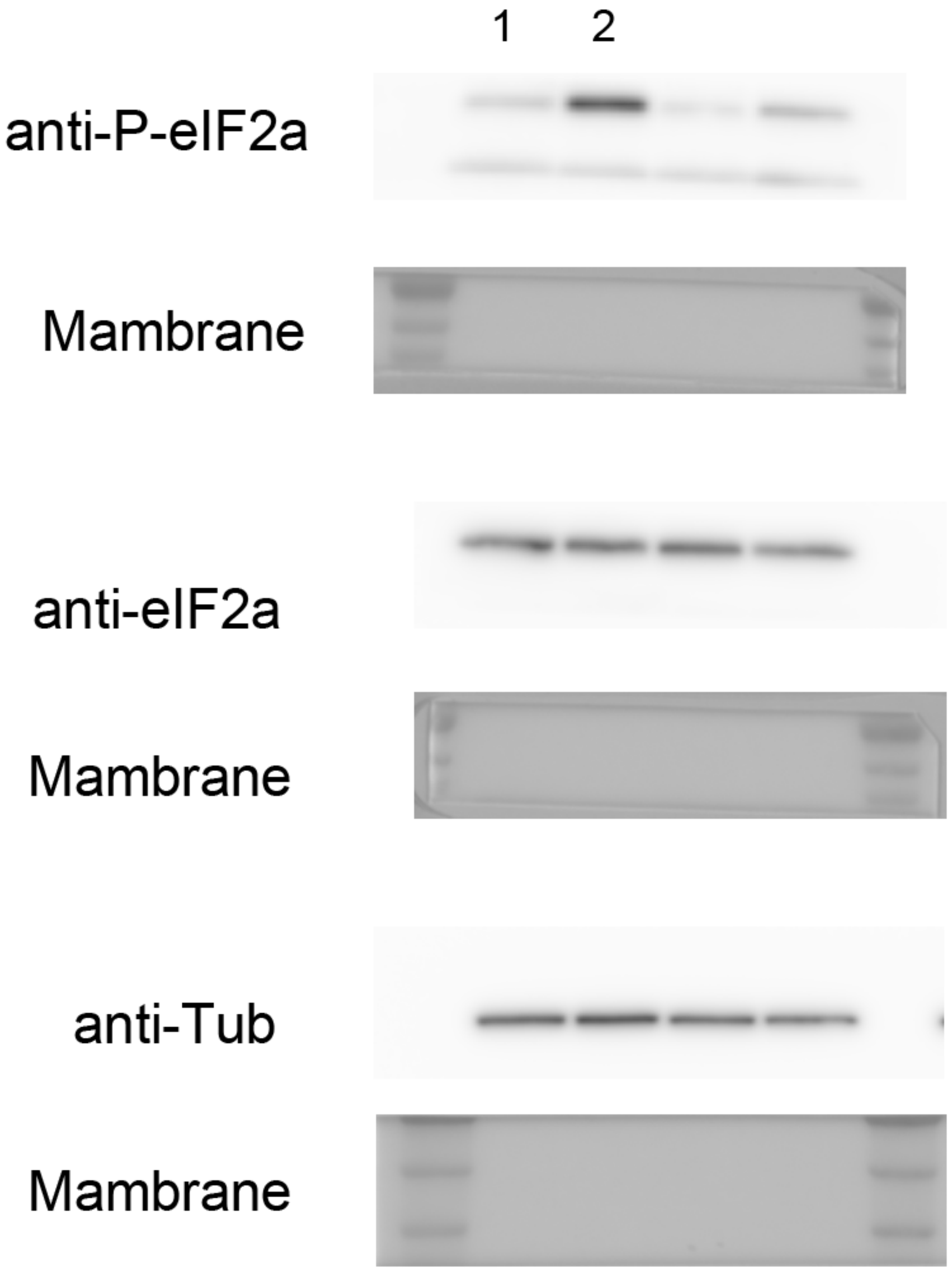

Figure 3B raw data

Lane1: wild type, Lane2: MZznf598, Lane3: +full, Lane4 +delta-RING

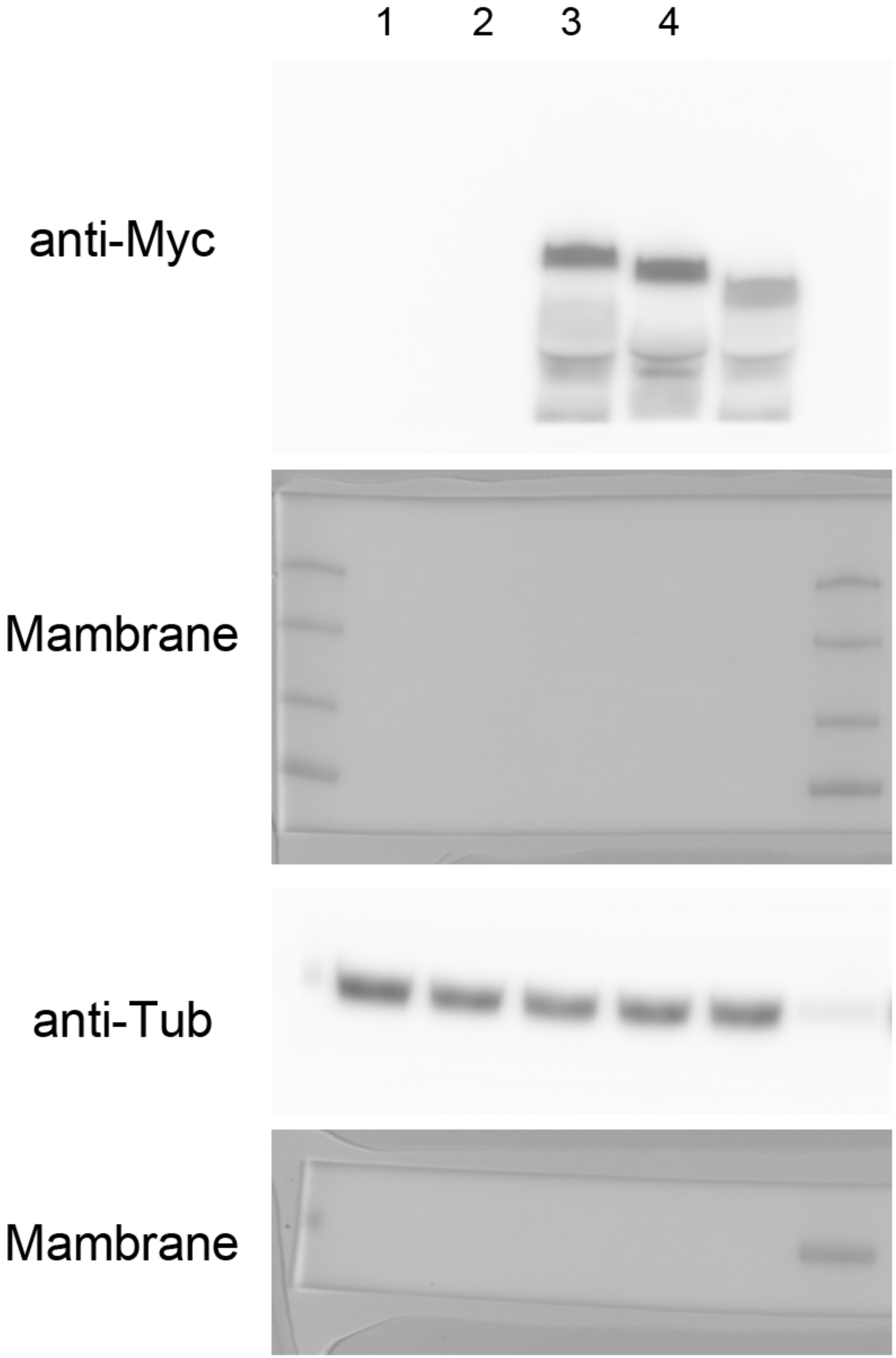

Figure 3F raw data

Lane1: wild type, Lane2: MZznf598, Lane3: A0

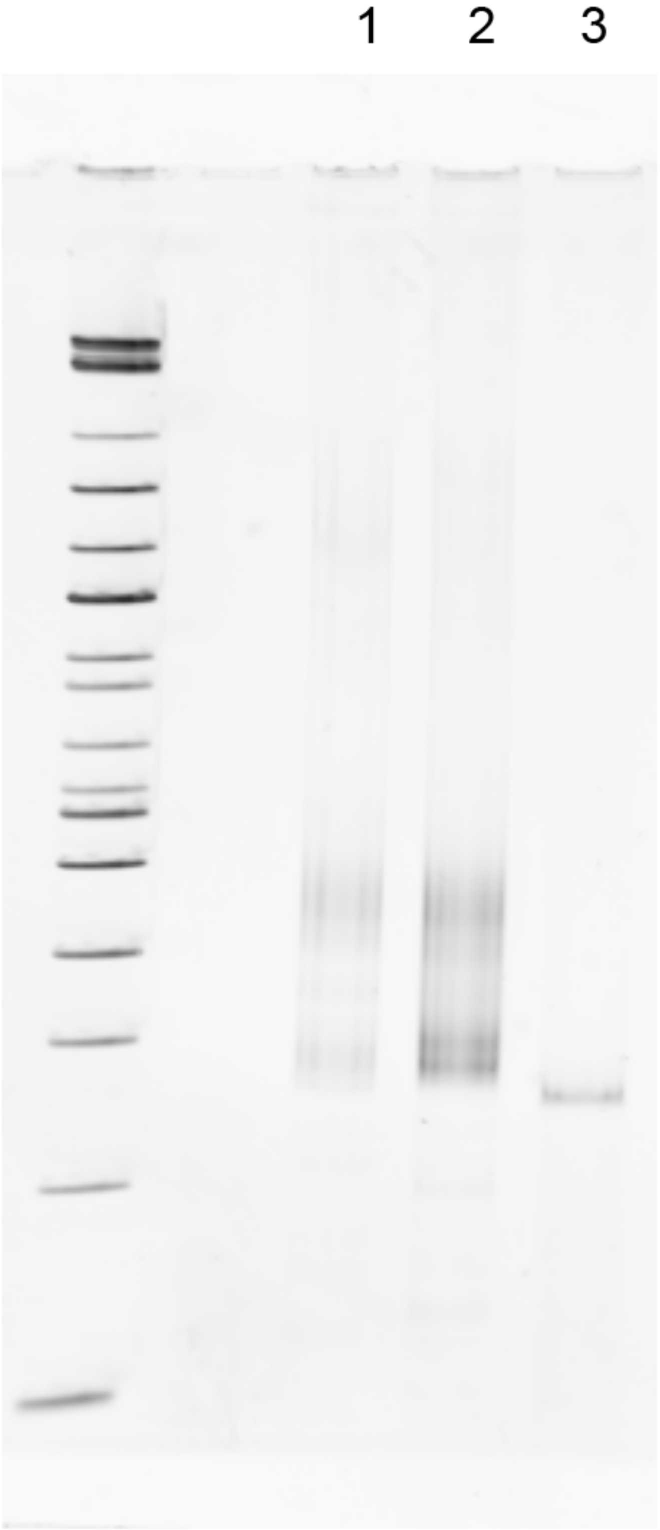

Figure 5E raw data

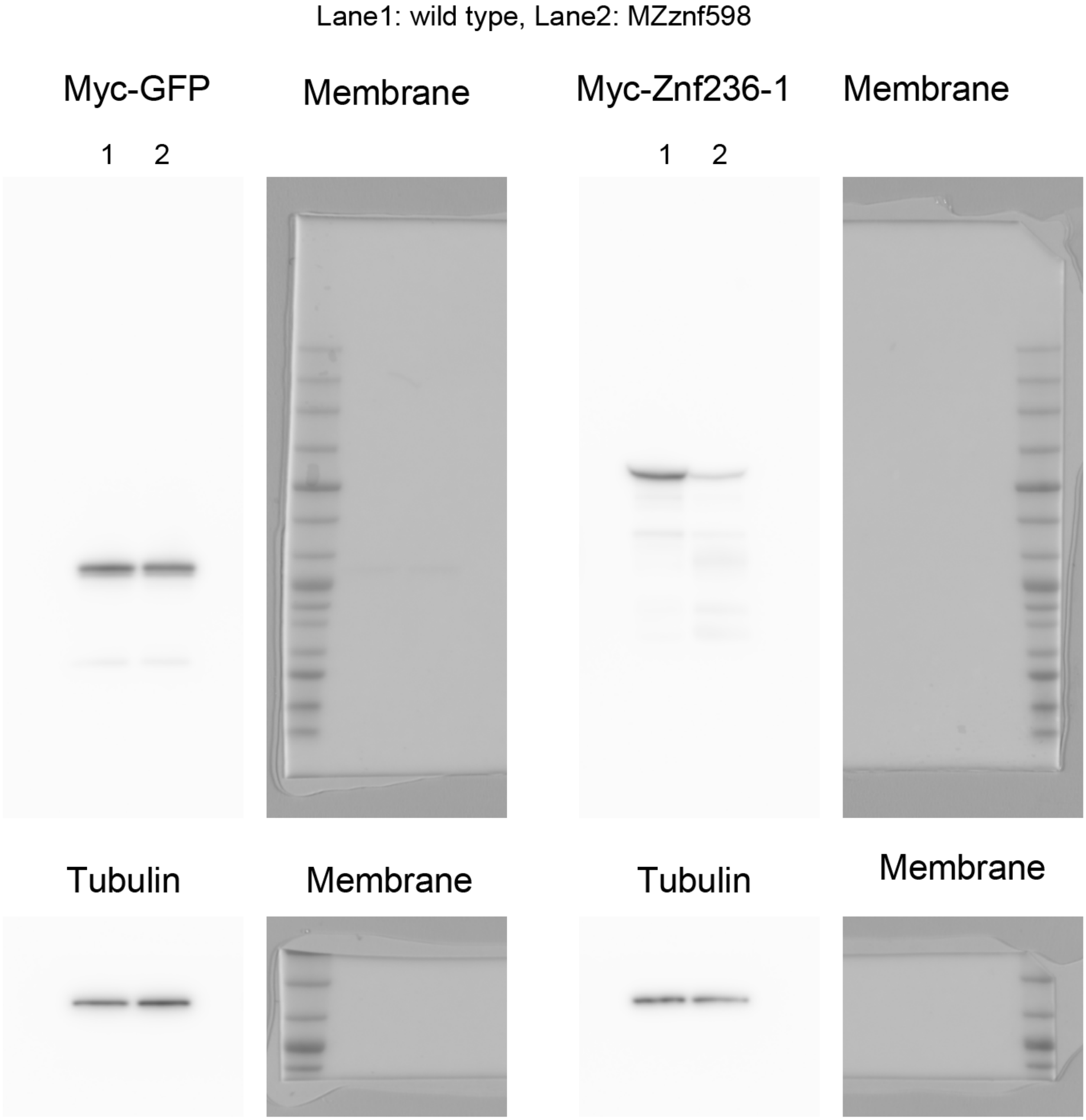

Supplemental Figure 7F raw data

Lane1: control KD, Lane2: ZNF598 KD

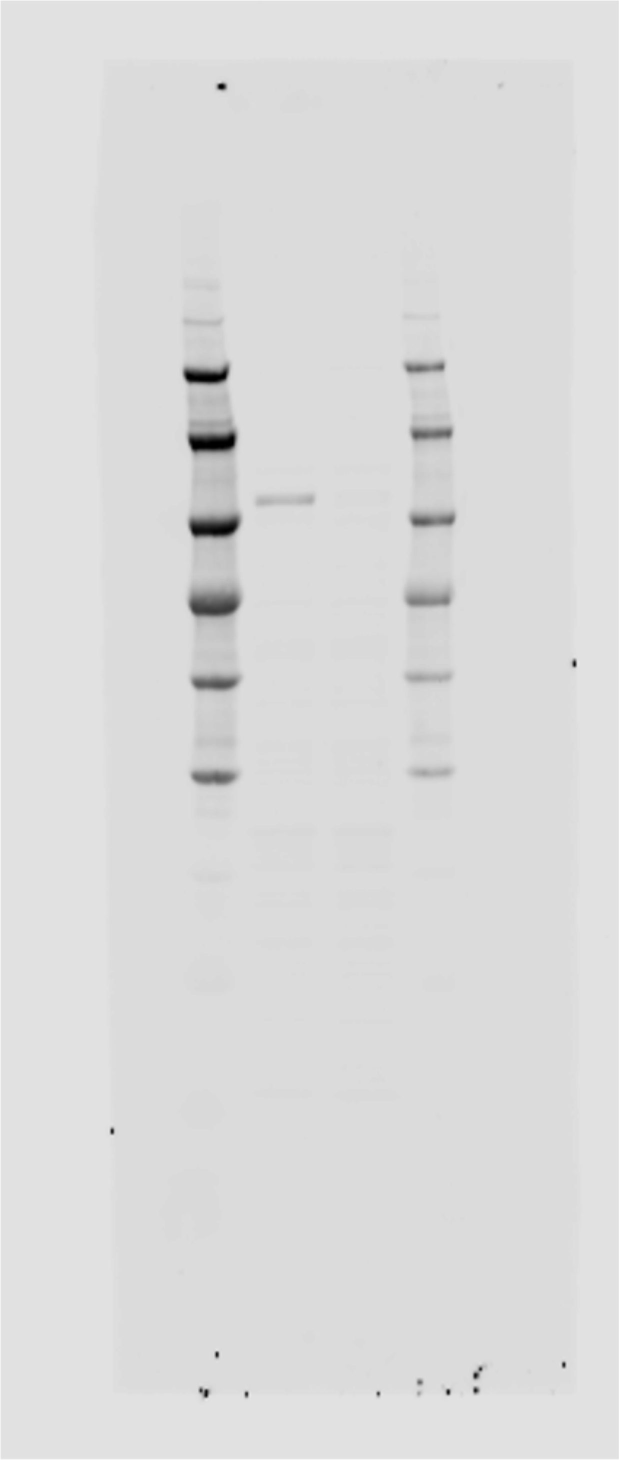

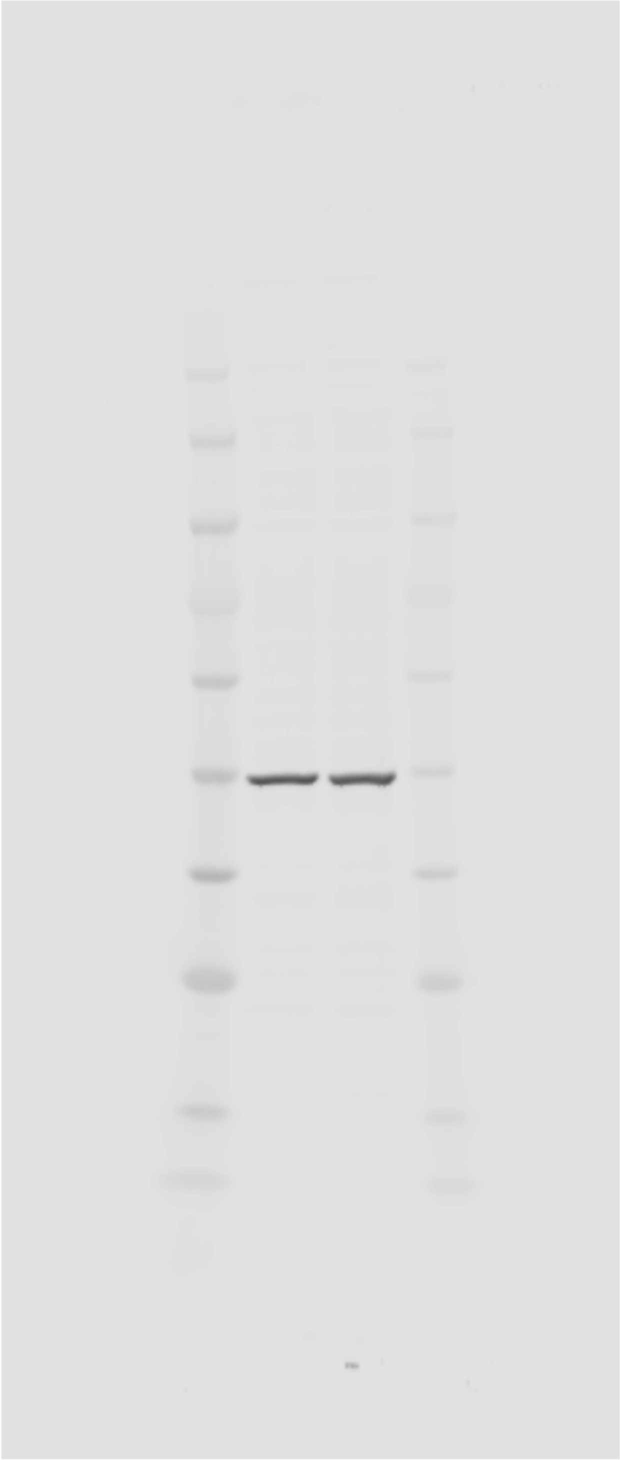

Supplement: S1 Raw Images — (PDF) [file pbio.3002887.s011.pdf]
