# Supplementary material for: Translation of zinc finger domains induces ribosome collision and Znf598-dependent mRNA decay in zebrafish
Source: PLoS Biol. 2024 Dec 5;22(12):e3002887. doi: 10.1371/journal.pbio.3002887 (PMC11620358; doi:10.1371/journal.pbio.3002887)
Supplement: Attachment — Submitted filename: Ishibashi_rebuttal.pdf [file pbio.3002887.s012.pdf]

Dear Reviewers,

Thank you very much for reviewing our manuscript, which is retitled “Translation of zinc finger domains induces ribosome collision and Znf598-dependent mRNA decay in zebrafish.” We are pleased to learn that the reviewers evaluated our study positively, and we thank the reviewers for their insightful and constructive comments.

We have performed additional experiments to address the reviewers’ concerns. In summary, 1) We conducted validation experiments for human C2H2-ZF mRNAs. While ribosome stalling at tandem C2H2-ZF sequences was confirmed in human cells, whether NGD is a primary determinant of C2H2-ZF mRNA stability in humans remained inconclusive. Therefore, we moved the human data to supplemental material and toned down the argument on humans to focus on zebrafish data. Accordingly, we changed the word "vertebrates" to "zebrafish" in the title. 2) To describe the abnormalities caused by the loss of Znf598 during embryogenesis, we showed the upregulation of phosphorylated eIF2α, an indication of the integrated stress response, during early embryogenesis. 3) We included additional controls and conditions in the ribosome stalling reporter analysis. We have revised our manuscript by incorporating the new data and suggestions from the reviewers. Please find our point-by-point responses below.

Reviewer #1:

1. Maternal zygotic ZNF598 mutant doesn't show apparent early embryonic development phenotype, suggest this gene is not important during early embryonic development and around 5 dpf authors start seeing reduced body length in mutant larvae. How would you correlate differentially upregulated candidate's gene contribution by RNA-seq at 6hpf (no phenotype) to 5 dpf phenotype and again becoming like wild type fish at later developmental stages.

We thank the reviewer for raising this important question. Although the morphological abnormality was not apparent, MZ*znf598* mutant embryos showed increased eIF2α phosphorylation (an indication of the integrated stress response) as reported in yeast and mammalian cultured cells (revised Figure 1B). One possible scenario is the cumulative effect of such translational stress starting in early embryogenesis emerges as a growth phenotype

at later stages. Related to this point, the presence of multiple mechanisms that act redundantly to solve or mitigate translation elongation defects, such as GCN1/2, ZAKα, and GTPBP1/2, might attenuate the phenotype in specific developmental stages, as we had discussed. Please also see our response to the reviewer 2.

Although we are eager to understand the molecular mechanism behind the growth phenotype, connecting specific target mRNAs to the phenotype requires much more work. Therefore, we would like to report the detailed embryonic and post-embryonic phenotype of the *znf598* mutant in our future study. We modified the related sentence in the discussion, as shown below, to highlight the importance of deciphering the impact of the Znf598 loss on target mRNAs in embryonic and post-embryonic stages.

Understanding the impact of ribosome collision events in each C2H2-ZF sequence on protein output and phenotypic outcome <u>in embryonic and post-embryonic stages</u> is an important topic for future studies.

2. Authors have shown how C2H2 domain containing genes gets differentially upregulated in ZNF598 mutant background in zebrafish and they have nicely validated some of these candidates by QRT-PCR, rescue experiments, morpholinos by blocking the translation and finally by RNA decay experiments. They did similar kind of experiment in human HEK cell lines and claim that this process seems to be conserved among different vertebrates but if would be nice if they can validate few of these candidates in cell lines and show similar trends of ribosome collision followed by RNA decay.

We thank the reviewer for suggesting these important experiments. To perform validation experiments, we selected three C2H2-ZF genes whose mRNA half-lives were elongated in the BRIC-Seq analysis under ZNF598 knockdown in HEK293T cells (revised Supplemental Figure 7A-C). These mRNAs have disome-rich tandem C2H2-ZF regions, and two out of four such regions caused detectable ribosome stalling in the dual luciferase assay in HEK293T cells (revised Supplemental Figure 7D). This result confirmed that the stall-prone nature of C2H2-ZF is common in zebrafish and humans.

To validate BRIC-Seq results, we inhibited transcription by Actinomycin D and analyzed the subsequent mRNA decay by time-course qRT-PCR. In this experimental setup, all three C2H2-ZF mRNAs showed similar mRNA decay patterns in control and ZNF598 KD cells

(revised Supplemental Figure 7E). Therefore, although BRIC-Seq data suggested a genome-wide effect of ZNF598 knockdown on C2H2-ZF mRNAs in humans, whether C2H2-ZF mRNAs are exclusively destabilized via no-go decay in human cells remained inconclusive. We are curious about this inconsistency and think it requires further investigation with additional experimental approaches, such as measuring mRNA half-life with more sensitive approaches or performing reporter assays.

Given these results, we would like to focus on the zebrafish data in the current study as the first report of ribosome stalling and NGD induction at C2H2-ZF mRNAs. We moved human data to supplemental figures and modified the text accordingly to be more neutral about the conservation of this phenomenon in vertebrates. The title was changed to indicate that the main conclusion is based on zebrafish experiments. We think that human data still holds scientific value for readers, but we are also aware that human data may be inconclusive and reduce the clarity of the study. Therefore, we would appreciate any suggestions from reviewers and editors on the human part.

We truly appreciate the reviewer's critical suggestions for performing these important validation experiments, which prevented the overstepped conclusion.

3. In almost all the experimental setup, authors have used proper controls and repeated experiments as required to run different statistical analysis, but it would be nice, if in figure 1 maternal zygotic ZNF598 zebrafish mutant experiments, authors should have sample size at least in double digits (N > 10).

We thank the reviewer for this suggestion. This comment is probably about Supplemental Figure 1D, as the data in Figure 1C (revised Figure 1D) already had a sample size with double-digit numbers (n=20). We obtained additional data for 12 and 16 weeks in Supplemental Figure 1D, and now the sample size is >10. We believe this additional data strengthened our initial conclusions. Due to the time constraint, the sample size is still below 10 for MZ*znf598* at 28 weeks. Therefore, we modified the text below to weaken the statement about data at 28 weeks.

MZznf598 fish <u>apparently</u> caught up with wild-type fish in the body length by 28 weeks after birth.

4. Figure2 C: In legend color name should be orange instead of pink. Also please check other colors name in different panels and in their respective legends.

We apologize for the inconsistencies. We checked and corrected the figures and legends carefully.

Reviewer #2:

1) The data in Figure 3E is confusing. Studies in yeast and mammalian cells have shown that deletion of Hel2/ZNF598 leads to readthrough of stall sequences. Yet, the data in this figure show the opposite. There is much less full-length product in the absence of Znf598, even though the mRNA is supposedly stabilized.

We agree with this reviewer's comment. The result was indeed an opposite to what we expected from previous studies. Having this reviewer’s comment, we tested if this opposite effect is a general consequence of ribosome stalling in zebrafish embryos (Figure 5J). When we performed dual luciferase reporter assay in MZ*znf598* embryos, tandem C2H2-ZFs of Znf236 caused stronger stalling in MZ*znf598* embryos than in wild-type embryos, consistent with the western blotting in Figure 5E. In contrast, tandem C2H2-ZFs derived from two FZNF genes showed reduced stalling in MZ*znf598* embryos, consistent with the yeast and human studies as mentioned by the reviewer. As we noted in discussion, both outcomes (roadblock or readthrough) could happen theoretically. We speculate that fates of stalled ribosomes in the absence of the quality control system are variable depending on the sequence context and kinetical parameters, such as duration, frequency, and plasticity of the stall. Other mechanisms acting on collided disomes, such as GCN2 and ZAKα, may also affect the fate of stalled ribosomes. Although interesting, clarifying these differences requires further biochemical and structural studies. The sequence variety of stall-prone tandem C2H2-ZFs will serve as good examples for conducting such future studies. Therefore, we would like to mention the possibilities of both cases in this study. We appreciate the reviewer's careful examination of our data.

2) In the same figure, it seems that addition of any fragment from Znf236 results in some stalling. These are missing proper controls. Have the authors tried control sequences from unaffected mRNAs? They would have to be of similar lengths.

We thank the reviewer for suggesting these important controls. In response to this comment, we included additional controls in Figure 5, either a fragment of LacZ or EGFP, which were similar in length to the tested sequences. These controls indeed simplified the data interpretation. For the longer Znf236 fragments (fragments 1-4), only fragment 1 caused

significant stalling compared to the LacZ fragment in a similar length. The same applies to the shorter Znf236 fragments (fragments 1A-D) and ZF repeats (ZF6x3, 7x3, and 8x3). All these data supported the original conclusion that ZF6 in fragment 1B is a primary cause of ribosome stalling.

3) If I understood it correctly, the embryo development in the Znf598 mutant cells appears normal. If that is true, what is the biological implication of having C2H2-containing mRNA destabilized by RQC? If these are real targets, why do not they appear to be important?

As this reviewer pointed out, MZ*znf598* embryos are morphologically normal. However, mutant embryos have defects at the molecular level because they show increased eIF2α phosphorylation (an indication of the integrated stress response), as reported in yeast and humans (revised Figure 1B). This may cause growth phenotype in post-embryonic stages. Related to this point, the presence of multiple mechanisms that act redundantly to solve or mitigate translation elongation defects, such as GCN1/2, ZAKα, and GTPBP1/2, might attenuate the phenotype in early stages and adulthood.

We think that the accumulation of ribosome collisions on multiple mRNAs causes harmful effects, and C2H2-ZF mRNAs are a class of such mRNAs. It is also possible that misregulation of FZNF, which is thought to be involved in transposon silencing, may cause genetic alterations on a longer timescale, as described in the discussion. Proving these models is indeed our future aim. Please also see our response to the reviewer 1. We appreciate the reviewer for his/her insightful comment.

4) I found some of the authors' arguments to be unjustified. For example, the statement "Comprehensive identification of the endogenous NGD target mRNAs is needed to understand the physiological role of NGD." By their nature, NGD and RQC are quality control processes that target aberrant RNAs, and the idea that they need to have endogenous targets is not accurate. Similarly, the statement "Chemically damaged mRNAs induce ribosome stalling and are potential NGD substrates; however, the frequency of such damage occurring on endogenous mRNAs is unclear" is unjustified. Cells evolved pathways to repair DNA and degrade damaged proteins, for example. The levels of damaged biological molecules might be low in a laboratory conditions, but under stress and in the presence of

chemical agents, their levels are known to go up.

We agree with this reviewer and apologize for the inaccurate statements. For the first point, we modified the sentence as below.

"*Comprehensive identification of the endogenous NGD target mRNAs is needed to understand the physiological role of NGD besides its established role in suppressing transiently generated aberrant mRNAs.*"

For the second point, we revised the corresponding sentence as below.

"Chemically damaged mRNAs induce ribosome stalling and are potential NGD substrates; however, endogenous mRNAs that are constitutively degraded via NGD under normal conditions remains unclear."

We hope these changes increase the clarity and accuracy of our statements. We thank the reviewer for the critical reading of our manuscript.

Reviewer #3:

1. The authors have shown that C2H2-ZF mRNA are stall- and collision-prone and degraded in a Znf598-dependent manner. RNA-seq analysis at different stages revealed significant changes of many genes in MZznf598 (Figure 1D), likely reflecting the role of Znf598 in other biological processes and/or secondary effects. Direct evidence for C2H2-ZF mRNA being NGD substrates is missing. The author should test whether ablation of the conserved homolog of Cue2/NONU-1, N4BP2, can rescue the abundance of these endogenous mRNAs and the reporter mRNAs. In addition, a hallmark of NGD is endonucleolytic cleavage. The authors should also examine whether decay intermediates could be captured using their reporters.

We thank the reviewer for this critical comment. We agree with this reviewer and are eager to test the effect of N4BP2 on C2H2-ZF mRNAs, but generating a novel mutant strain still requires a significant amount of time in vertebrates. Instead, we tried overexpression of N4BP2 in zebrafish embryos. However, the effect of N4BP2 overexpression on Znf598 target mRNAs was unclear, partly because these mRNAs were already unstable in wild-type embryos.

While N4BP2 is considered a Cue2/NONU-1 homolog and is indeed recruited to collided ribosomes (Shinha et al., eLife. 2020), it has not been shown that N4BP2 mediates the endonucleolytic cleavage in vertebrate NGD. We also would like to mention that loss of Cue2 alone does not sufficiently stabilize NGD target mRNAs in yeast, probably because there is an alternative mRNA decay mechanism mediated by Syh1 and Smy2, yeast homologs of vertebrate GIGYF1/2 (D'Orazio et al., eLife. 2019; Veltri et al., eLife. 2022). Considering these points, we feel that validating the role of N4BP2 in vertebrate NGD holds significance on its own and is worth reporting as an independent study.

To address this reviewer's comments, we utilized the sfGFP reporter mRNA with 1B fragment of *znf236* used in Figure 5 and performed time-course northern blotting to test if NGD cleavage product was detectable in zebrafish embryos. However, we could not detect cleavage product clearly (please see the figure below). This result was well expected from yeast studies because the NGD cleavage products were barely detectable unless exonucleases were depleted.

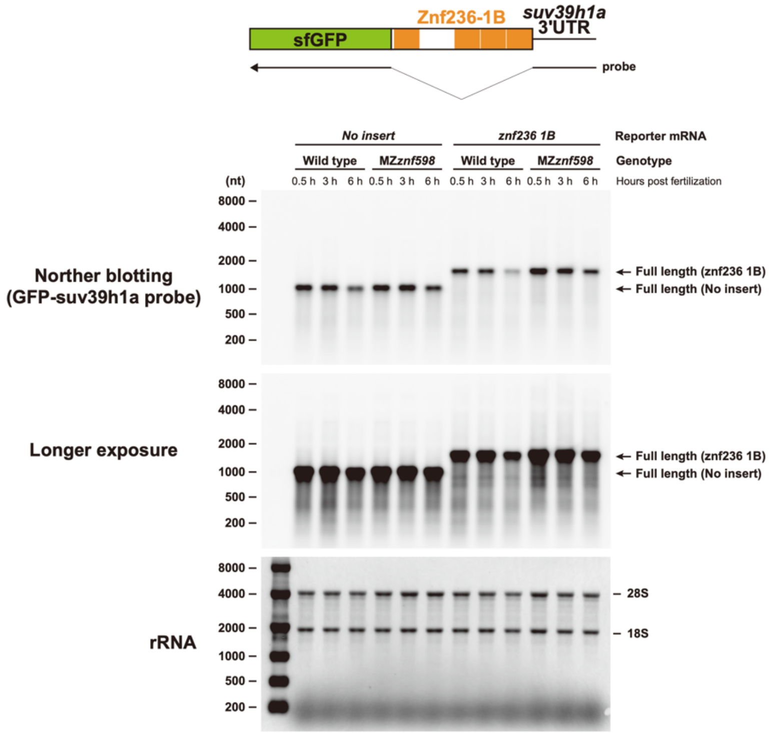

To seek other evidence, we utilized our Rps10 mutant zebrafish strain, where lysine residues ubiquitinated by Znf598 were substituted with arginine (Rps10 KR mutant, Ugajin et al., RNA 2023). Since this mutant still has intact Znf598 target ribosomal proteins known to be ubiquitinated in yeast and humans – such as Rps20/uS10 and Rack1/Asc1 – we expected a partial loss of the NGD activity in Rps10 KR mutant embryos. Indeed, we observed mild but significant stabilization of *znf236* mRNA in maternal-zygotic Rps10 KR mutant embryos (MZ*rps10*KR) (Supplemental Figure 2E). This data supports the argument that ribosome stalling at C2H2-ZF sequences induces mRNA decay via ribosome ubiquitination by Znf598.

Characterization of NGD in vertebrates still requires a lot of work compared to yeast and worms. We believe that our study will contribute to understanding NGD in vertebrates further. We carefully revised the manuscript to avoid overstatements and to mention the ambiguity of vertebrate NGD in discussion as below.

Information on NGD and its substrate mRNA is still limited in vertebrates compared to yeast and worms. In future studies, C2H2-ZF mRNA will be a good model for characterizing NGD in vertebrates.

2. Complementary to Specific point 1 above, a key feature of RQC is CAT-tailing. If these C2H2-ZF are endogenous substrates of RQC, they should undergo CAT-tailing. It may be challenging to monitor some of these endogenous proteins by immunoblotting due to their low abundance. However, the authors have established a reporter system using these stall-prone sequences. Such a setup could be used to examine CAT-tailing.

We agree with this reviewer that detecting CAT-tailing will strengthen the evidence for ribosome stalling and RQC at the C2H2-ZF sequence. Therefore, we constructed a new reporter mRNA containing the stall-prone 1B region of Znf236 placed between Myc-tagged EGFP and LacZ fragment to detect truncated products stalled within the 1B region. We injected this mRNA into wild-type and MZ*znf598* embryos and detected the Myc-tagged products (please see the figure below). While the truncated products presumably generated by ribosome stall in 1B region were observed, identifying the CAT-tailing product was difficult for the following reasons.

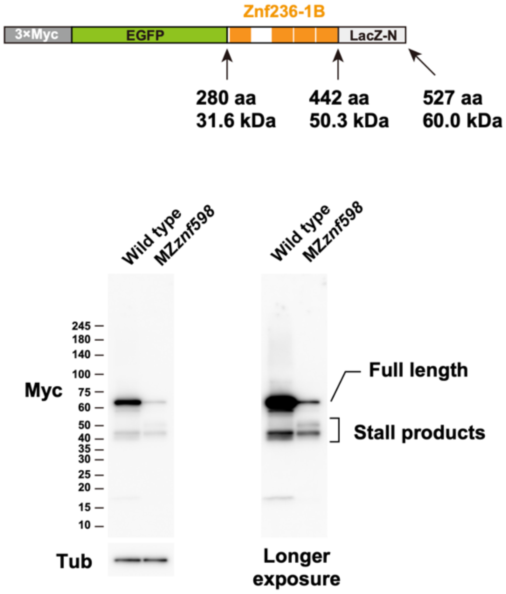

1) Considering the disome profiling data and western blotting signal, ribosome stalling probably occurs at multiple positions across C2H2-ZF repeats. As predicted by the reviewer, it was difficult to distinguish a relatively small amount of CAT-tailing products from other stall

products, which are slightly different lengths. 2) Due to its instability, the detection of CAT-tailing requires Ltn1 depletion or proteasome inhibition. As the Ltn1 mutant is unavailable in zebrafish, we tried the latter using MG132. However, MG132 was toxic to zebrafish embryos and caused a significant reduction of reporter expression, making it difficult to obtain reliable data.

We are happy to include the data above if the reviewers find it necessary. In our future study, we plan to conduct biochemical analysis using the C2H2-ZF sequences as model substrates of endogenous RQC and NGD targets, which are still limited in vertebrates. We truly appreciate this reviewer's comments.
